# Cancer-related concerns and needs among young adults and children on cancer treatment in Tanzania: a qualitative study

**DOI:** 10.1186/s12885-019-5279-z

**Published:** 2019-01-17

**Authors:** Thecla W. Kohi, Louise von Essen, Golden M. Masika, Maria Gottvall, Justine Dol

**Affiliations:** 1 School of Nursing, Muhimbili University of Health and Allied Sciences, Dar es Salaam, Tanzania; 2 Clinical Psychology in Healthcare, Department of Women and Children’s Health, Uppsala University, Uppsala, Sweden; 3 Nethersole School of Nursing, Faculty of Medicine, The Chinese University of Hong Kong, Shatin, Hong Kong; 4 School of Nursing and Public Health, The University of Dodoma, Dodoma, Tanzania; 5 Department of Health Sciences, The Swedish Red Cross University College, Huddinge, Sweden; 6 Faculty of Health, Dalhousie University, Halifax, Canada

**Keywords:** Cancer, Children, Young adults, Cancer treatment, Tanzania

## Abstract

**Background:**

Cancer is one of the leading causes of morbidity and mortality worldwide. Seventy percent of deaths of cancer occur in low or middle-income countries, where the resources to provide cancer treatment and care are minimal. Tanzania currently has very inadequate facilities for cancer treatment as there are only five sites, some with limited services; two are in Dar es Salaam and one each in Mwanza, Kilimanjaro and Mbeya that offer cancer treatment. Despite cancer being a prevalent problem in Tanzania, there is a significant shortage of information on the experiences of young people who receive cancer treatment and care. The aim of this study was to explore cancer-related concerns and needs of care and support among young adults and children who are receiving cancer treatment in Dar es Salaam, Tanzania.

**Methods:**

Using an explorative, qualitative design, two focus group discussions (FGDs) with young adults (18 to 25 years) and four FGDs with children (9 to 17 years) were held. Data were transcribed into English and analyzed using content analysis.

**Results:**

Identified concerns included physical effects, emotional effects, financial impacts, poor early care, and poor treatment. Identified needs included the need for improved care in hospital by the staff, need for community support, financial needs, needs for improved cancer care and treatment in the hospitals, and the need for increased education about cancer. Resilience was identified, particularly around hope or faith, having hope to be healed, and receiving good care from staff.

**Conclusion:**

Young adults and children receiving cancer treatment in Tanzania have many needs and concerns. Improvements with regard to the care provided in hospital by the staff, the cancer care and treatment in the hospital, and population-wide education about cancer are necessary to address the identified needs and concerns. Further studies on specific approaches to address the concerns and needs are also warranted.

## Background

Cancer is a common health threat all over the world. According to the World Health Organization, cancer is one of the leading causes of morbidity and mortality worldwide [1]. In 2018, there were approximately 9.6 million deaths, with about one in every six people dying from cancer-related causes [1]. Cancer is the second leading cause of death for all ages around the world, but the leading cause of death for children and adolescents aged 0 to 19 years [1, 2]. A large proportion (70%) of these deaths occur in low or middle-income countries, where the resources to provide cancer treatment and care are minimal [3]. The cancer burden in these countries can be reduced through early detection and effective treatment management yet challenges remain in many low and middle-income countries. Currently in Tanzania, cancer services are offered in five centres: two are in Dar es Salaam [4, 5] one in Mwanza [6], one in Kilimanjaro [7] and one in Mbeya [8]. However, they are inadequate to cover the need for cancer treatment as only one site located in Dar es Salaam offers a wide range of services including diagnostic, chemotherapy and radiotherapy, while the rest offer only limited services [5]. In 2014, there were approximately 21,000 reported deaths from cancer in Tanzania and 35,000 new diagnoses across all ages [9]. While the World Health Organization estimates that the overall incidence of childhood cancers is approximately 300,000 children per year [10], there is a paucity of data on the incidence of childhood cancer diagnosis in Tanzania and sub-Saharan Africa more broadly [11]. Once Tanzanian patients have been diagnosed with cancer, they are eligible for free treatment, but patients must pay for screening out of pocket [9], which can potentially lead to cancer-related deaths to go undiagnosed and unreported.

In addition to the challenges related to diagnosis and treatment, children and young adults with cancer experience stressful physical and emotional suffering that could significantly affect their well-being including lack of self-esteem, physical incapacity, and poor educational progress [12]. While on treatment, children experience pain, nausea and vomiting, fatigue, weakness, loss of hair, loss of limb function and attention deficit problems, all of which amplify fear, anxiety and depression [13, 14]. Moreover, stressors related to cancer in children and young adults can affect the whole family and lead to separation of siblings, family loss of emotional control, disruption of family routines, fear, extended family conflicts, and impact on financial strains [12, 13].

Healthcare providers need to understand the physical and psychosocial experiences of children and young adults with cancer to appropriately address their challenges while they receive cancer treatment. Healthcare providers are not always trained in psychosocial support as a component of quality cancer care, and thereby fail to manage the psychosocial symptoms that patients present with, despite the call for improved training in healthcare providers [15]. If psychosocial needs of children and young adults with cancer are not met, it is likely that negative effects of cancer including fear of death, depression, lower pain tolerance and disability, among others, will increase [12]. This may lead to unnecessary, repeated, or frequent hospitalization or intrusive procedures and lower quality of life [16, 17].

Despite cancer being a prevalent problem in Tanzania, there is currently a paucity of knowledge on the experiences of Tanzanian children and young adults who receive cancer treatment and care. While only a few studies have examined the psychosocial situation of Tanzanian cancer patients [18–20], some of which are over a decade old, none of these focused on concerns or needs of care and support for children and young adults,. Consequently, the lack of information makes it difficult for stakeholders, including the government, partners, and the community, to provide appropriate care and support [21, 22]. This study aims at reducing this knowledge gap.

### Aim of the study

The aim was to explore cancer-related concerns and needs of care and support among young adults and children who are receiving cancer treatment in Dar es Salaam, Tanzania.

## Methods

### Design

Using an explorative, qualitative design, focus group discussions (FGDs) were held to explore cancer-related concerns and needs of care and support among young adults and children. Focus group methodology was chosen based on its ability to facilitate semi-structured conversation between participants to explore individual and collective experiences [23].

### Setting and population

Young adults (18 to 25 years) and children (9 to 17 years) were recruited. Young adults were recruited from Ocean Road Cancer Institute (ORCI), a hospital in Dar es Salaam, Tanzania where the majority of Tanzanian adults with cancer receive care and treatment, accommodating approximately 250 patients [5]. Children were recruited from the paediatric cancer centre at the Muhimbili National Hospital (MNH), in Dar es Salaam, Tanzania. The centre accommodates approximately 50 children [4].

### Procedure and data collection

Six FGDs were held with 22 participants comprising of two groups of young adults and four groups of children respectively, split by sex. An interview-guide was developed and piloted with the guide modified based on the initial feedback from the participants (see Table 1 for the questions). The questions were open-ended and explorative, developed with the aim to explore an understudied subject. Given that there is a lack of knowledge about the situation for young cancer patients in Tanzania, questions around topics that would be useful to inform about cancer care for the population and ways to improve that could be posed to young adults as well as children were developed. The same questions were posed to all participants and the moderator was allowed to clarify the content of questions if needed. However the questions did not appear to be difficult for any participant, including the younger ones and clarifications were not needed.

**Table 1:** Focus group interview guide

Questions	
1. Has anything bothered you because you have cancer?	
**Probes**: If so, what has bothered you? In the hospital? Outside the hospital? Before? Now?	
2. Has your cancer disease affected your life?	
**Probe**: If so, how?	
3. Could the hospital and the hospital staff do anything to help you?	
**Probes**: If so, what could they do? Before? Now?	
4. Could anyone outside the hospital do anything to help you?	
**Probes**: If so, what could they do? Before? Now?	
5. What is your view of the future?	

The inclusion criteria included speaking Kiswahili fluently and being in sufficient physical condition to participate, as recommended by the nurses in-charge of the respective wards. The same nurses were responsible for recruiting young adults and children to the FGDs. Ethical approval was obtained from The National Institute of Medical Research (NIMR) in Tanzania with permission to conduct the study granted by ORCI and MNH prior to study-start in September 2015.

During FGDs, participants sat comfortably at a round table to facilitate interaction with the moderator and observer present who sat among participants. Before the respective FGD, participants were informed about the study orally and in writing and completed consent/assent forms. For the children, one guardian per child provided written consent for the child to participate with the child providing written assent. Data collection began with completion of a questionnaire about participants’ demographic data. At the beginning of the FGDs, participants were reminded of the voluntary nature of participation and that they should feel free to withdraw at any time. A moderator (GM) led the discussion while an observer (research assistant) took notes. FGDs were held at ORCI and MNH respectively, were audiotaped and conducted in Kiswahili. Each FGD took approximately 60 min.

### Data analysis

Descriptive statistics were used to summarize participants’ demographic characteristics. The recordings from the FGDs were transcribed verbatim by a transcriber fluent in Kiswahili and English. The transcripts were verified for accuracy against the interview recordings by the co-authors GM and TK who are fluent in Kiswahili and English. The transcripts were then translated into English by the same person who transcribed the recordings and were again checked for accuracy by GM and TK. Data were analyzed using content analysis whereby the entire transcribed text was read and notes of what was said in the FGDs were made in the margins, identifying codes [24, 25]. The codes were collected and reviewed, with similar codes grouped together in themes. The transcripts were read again to ensure that all codes had been identified and that data that fit under a certain theme were labeled as necessary [24]. This was led by co-authors JD and TK and then reviewed and confirmed with co-authors GM, LvE, and MG.

## Results

### Participants

A total of 22 participants participated in the six FGDs, eight young adults (50% males) and 14 children (57% boys). Young adults were on average 21.0 years (SD = 2.6) whereas children were on average 13.3 years (S = 2.3). Both young adults and children lived an average of 725 km from the hospital (range: 60–1300 km). Young adults were diagnosed with blood cancer (*n* = 2), cervical cancer (*n* = 2), and one each of: ear cancer, skin cancer, eye cancer, and cancer of the glands. Children were diagnosed with blood cancer (*n* = 6) and cancer of the glands (*n* = 4), followed by one each of: kidney cancer, liver cancer, foot cancer, and undiagnosed. All children had a guardian with them and a majority of their guardians were mothers (71%), whereas only 38% of young adults had a guardian with them, all were fathers. All except one young adult did not know their stage of cancer (96%). Participants were either on chemotherapy (*n* = 14), chemotherapy and radiation (*n* = 3), chemotherapy and surgery (*n* = 4), or radiation (*n* = 1).

### Concerns

Five themes emerged related to concerns experienced, including physical effects of cancer, emotional effects of cancer, financial impacts, poor early cancer care, and poor cancer treatment.

#### Physical effects of Cancer

Several aspects emerged related to physical effects of cancer, including pain associated with cancer; loss of ability to do things as before, like going to church, participating in sports, playing and working, and interruptions to schooling and/or delayed education. For instance, related to the physical pain, one female child said “… I cannot walk … I cannot sleep at night because my stomach is aching” and another said: “Trouble is, the pain, the body hurts all the time”. Another female child expressed:

Now, even when my relatives wash dishes, I feel like I cannot do it. When they wash clothes, I can't join them because I get tired when I touch one cloth I say to them ‘I’m tired let me have a bit of rest', which makes me feel bad when I see others can do this and I can't.

Other aspects related to the physical impact of cancer included being physically sick (vomiting) or experiencing negative side effects of medication. Participants said: “After I started using this medicine, it caused vomiting. Sometimes I get discomfort and I vomit, when I take the medicine” [male child]). Young adults also mentioned a loss of strength and/or energy (“I used to work hard most of the time [but] I became tired easily and I found myself weak. It was really a challenge” [male young adult]) and children expressed having poor health (“My health has changed due to illness”).

#### Emotional effects of Cancer

Cancer treatment not only had physical effects but also emotional effects. Participants expressed concerns around the impact on their family, a male child said:

For it is a long process, perhaps many arrangements at home fail. For what it was, we as children with cancer have difficulty learning, which is a problem. The parents as well find it difficult to bring you [to the hospital] and then you look at the income itself, the rest of the brothers need to attend the hospital. Over three years, it becomes a problem.

Other emotional effects include missing home while receiving treatment (“I often remember home”), negative feelings about diagnosis and/or a loss of hope in recovery (“I felt that if I was admitted again, there shall be no return”), fearing death (“…sick from cancer, you will not [live] if you go there, you’re going to die alone”) and loss of one-self due to restrictions (“…sickness changes you to be different from how you were, there are certain things you are told not to use even if you recover, it can come back”).

Young adults expressed experiencing stigmatization, with a male young adult explaining:

I was stigmatized…because those suffering from blood cancer are thin, similar to people suffering from HIV…so we Tanzanians, many are doctors by eyes so when he sees without testing you, he starts to spread rumors in the street, that a particular person is a victim [of HIV].

The issue of infertility due to operation of the uterus was a concern for female young adults. One young adult said; “On my side they operated me, and they totally removed the uterus. Now, that has affected me a lot because I will no longer be able to give birth”.

#### Financial impacts

Many participants expressed concerns around the financial impacts of cancer treatment, including that medication and/or treatment is expensive and that they were unable to receive full treatment due to lack of funds and inadequate equipment. One male young adult said:

You are prescribed [chemotherapy] drugs which cost eight hundred thousand [TSH] or three hundred thousand, but you don't have the ability to purchase those drugs. You have to go around and seek for those drugs, and if you don't find, you have to go home. And then your condition becomes worse.

Likewise, a female child said:

…one day, my parents were told certain injections were going to cost almost millions [TSH]. It was nearly two million for 24 injections until I would finish the drugs. I have been given eight injections only. Now, other injections remain but I have no money and now we were supposed to get money to finish up.

Treatment for cancer in Tanzania is supposed to be covered by the government once a patient is diagnosed. However, the male young adults mentioned that treatment is supposed to be free, but it is not:

When I first came here, I was told the treatment is free, the medicine is free, food is free, and you don't even pay for hospitalization. In contrary, when I came here, the second day when I was seen by the doctor, I was prescribed and told to go outside to buy medicine. You find that my dose costs five hundred and fifty thousand [TSH] for each dose, and I have to take it twelve times, so there is a small possibility of me getting them all.

Male young adults reported that they often had to pay bribes to receive treatment: “When the doctor comes to talk to you, you had to give money, which is bribes, so that he can treat you” and “you need to bribe the doctor if you want your issue handled quickly. If you come empty handed, you will be told to go home, and your condition gets much worse”. Patients reported ethical concerns related to the care provided by the healthcare staff. For example, one young adult said: “I would ask for a commission to be created, or people from nursing schools to be well trained. A new commission to trap people who give bribes that may help to reduce the problem. But also, to ask more doctors to strive to provide services for patient as required”.

#### Poor early Cancer care

One of the greatest challenges identified was associated with wrong diagnosis and/or treatments when patients first seek care. A female young adult said “In our hospitals, they do not use tests. They were just treating; they give you medicine only. Many years I used drugs but without success”. A male child explained his story about diagnosis as:

When we came [to the hospital], they sent us to the ward of children with sickle cell. They investigated, and the results showed no sickle cell. But they gave me medication and after the drugs, the swelling of my lymph nodes went down, so they let me go back home. When I went home, I stayed one week, and it started again. We came back here again, they examined me and found no sickle cell...finally, they tested my bone marrow and when the results came, it showed signs of cancer.

Participants expressed that there was a delay in initiation and continuing treatment as well as a delay in diagnosis and results, that treatment was provided far from home, that treatment was not available close to home and that they had had to transfer to referral hospital. A female young adult said:

At the hospital, they said 'we are not sure about this disease, but we shall give you these drugs. Go and buy and if you don't see any improvement, come back’. I started taking them, but the swelling remained; so the second month, we returned to the hospital. They said, we can't do the investigation here, so they referred me to Ocean Road.

#### Poor Cancer care

A final concern participants mentioned was the poor cancer care available once they were diagnosed and receiving treatment. They expressed that medications were not always available and that there was a lack of treatment: “sometimes, one can find no medicine. Patients who are arriving at the hospital are told to go buy medicine.” Male young adults complained about a lack of available pain medication, with one young adult expressing:

I suffer much pain and the pain becomes worse at night. If you tell the nurse that you want pain medication, you are told ‘no pain medication and the hospital pharmacy is closed, and we cannot give you any pain medication until you are seen by your doctor, so you have to sleep with your pain. Pain becomes more severe and it can’t cease.

Participants expressed receiving poor care at the hospital and that staff communicated poorly regarding treatment and care. One male young adult said about the care he received: “when I arrived at Muhimbili, I was given a bed. I stayed the whole week without any attention”. Likewise, a female child complained of poor communication about her treatment “when you go to ask (questions), you are given rude answers. Sometimes the results indicate the condition that you have but they ask questions and argue with you”.

Participants also mentioned poor living conditions at the hospital, with male children saying “…passing through many rooms, mosquito nets are torn, so you have difficulty fitting them, still mosquitos pass through the holes. So, while still in the hospital, you can sometimes get malaria”. A boy child complained: “Food is not cooked well. Sometimes you find sand in your food. Sometimes the beans are half cooked.”

Participants identified that equipment was often broken or lacking (“Muhimbili is a National Hospital but you go there, and you find no equipment”). Female young adults mentioned not having lights at their toilets and outside the toilets. Long waiting times for appointments were mentioned. A male young adult said: “You are sick, but you are given a long-standing appointment. You are told that there are other appointments [that other people have] so you have to wait. Maybe after a month and a half, then there will be a chance to see a doctor.”

### Needs

Six themes emerged related to needs including the need for improved care in hospital by the staff, need for love and compassionate care by hospital staff, need for community support, financial needs, needs for improved cancer care and treatment in the hospital, and the need for increased education about cancer.

#### Need for improved Care in the Hospital by the staff

A need identified by children and male young adults was for improved care provided at the hospital by staff, desiring for better quality care. For instance, one male child provided this example about poor care he received and what he would desire to see instead:

When you are given medicine [and] when the drip is finished, it should be removed as the parent is not supposed to touch it. Sometimes, you may have three drips to be changed and you may wait for the nurse, who comes late. So, you may stay with the drip [in your arm, finished]. And, if you stay with it for a long time, the blood starts to flow back, and the line might block and it is difficult to run another drip, which leads to inserting a new line. So, it becomes an inconvenience. It becomes a problem.

#### Need for love and compassionate care by hospital staff

Children expressed the need for love and compassionate care by the nurses and doctors: “[I would like doctors and nurses] to give me medicine and comfort…to look at me all the time to see how I am doing (boy child)”. A girl child said: “I only wish just nurses to have more compassion, …. there should be no disturbances”. Likewise, another male child said it would be more beneficial if healthcare providers were close to patients, “they [nurses and doctors] have to show love to the patient. …. they should run fast to the patients [in need] to listen and help.”

#### Need for community support

Participants expressed a need for support from the community. All reported a need for community and family support, particularly emotional support and through prayer. A male child said: “I wish them [community members] to pray for me to recover and come back like before”. Young adults expressed a need for support to get available treatment. A female young adult gave an example of support she received by saying: “when I got my results, I also went to the office of Social Welfare and they supported me with transport to come up here [to Ocean Road]”.

#### Financial needs

Not surprisingly, since one of the concerns was related to financial impact, all participants expressed a need for community support through financial support and the provision of food. A female child said: “We need support, that is, we are given a date to come, and we should come on the same day. Now, sometimes parents do not have money. We have to be given money, to support our transport to the hospital. Other treatments need money.” Likewise, a male child said: “[Community members can] help with other items, such as clothing and food. They can come to see us, they can comfort us and give us food”. A female young adult said: “People from outside, they provide a great help...they distribute many things that are very helpful, we are very grateful.”

#### Need for improved care and treatment in the hospital

Also in line with concerns expressed, participants reported a need for improved care and treatment while in the hospital. Children and young adults expressed a need for government to provide funds for cancer treatment and basic supplies for hospital. For instance, a male child said “I want to see the government boost the hospital pharmacy because sometimes one can find no medicine. Patients arrive at the hospital, and they are told to go buy medicine”. Likewise, a male young adult said: “I beg the government to work on this. They should help us find a way to get the chemotherapy and could help increase the number of machines for radiation because people suffer very much”.

Children and female young adults expressed a need to be able to access medication and treatment when needed: “All needs of patients in the hospital, due to their sickness, like treatment, drugs, and certain medications, should be available” (female child). Furthermore, equipment should be available when needed, as suggested by a female young adult:

They have to improve. They should buy radiotherapy machines. Even though they have two, one of them is not working so there is only one. And it is very difficult for a technician to come, so we ask that to be improved and increase the number of machines.

Interestingly, male young adults expressed the need for local cancer centers and information about the drugs they were receiving. Regarding the need for local centers, a male young adult said:

On the side of the government, I request them to establish special fund to support cancer patients and the radiation machine. They could prepare regional hospitals to cater for those services. Because we who live outside Dar es Salaam, we are far away from the place where cancer services are provided. For example, when a person is thinking about coming here, he starts thinking about the fare to come here.

A need for availability of blood for transfusion was mentioned. Participants with blood cancer needed blood for transfusion, however it seemed that there was no adequate supply of blood for everyone in need. Moreover, those who received it needed to pay to get blood transfused. For example, one male young adult said: “And if you come here you are told if the blood is low, blood is given for free. However, if you come here they ask you, if you need blood you have to pay thirty thousand Tanzanian schilling per drip, if you required sufficient blood, you will be required three drips and pay ninety thousand”.

#### Need for increased education about Cancer

Finally, young adults expressed a need for better education about cancer, cancer causes and signs of cancer for diagnosis, particularly in rural areas. For instance, a female young adult said:

If we went there [to the doctors], when we are sick, they should not give us drugs, because these drugs themselves will be increasingly toxic. Because they give you only medicine without doing any test. If you have finished, they give you some other. They don't test.

Additionally, a male young adult said related to the need to education on cancer in general:

I would like the community to know the causes of cancer, although there are some cancers which are inherited, for example blood cancer, some are caused by the particles in the mining. So, my thinking is to have them receive education about cancer as they receive about HIV.

### Resilience

While not a concern or need, a theme that emerged in the FGDs was the idea of resilience with both children and young adults expressing that hope had positively impacted their cancer-related experiences. Almost all groups, except for male young adults, expressed that their faith was helping them through the process. One female child said “without God, I would not have been here. And in normal circumstances, the way we are is because of God, so when we hear the words of God, we are consoled very much”, a female young adult said, “my belief gives me power, it gives strength of hope”.

Another aspect that emerged in all FGDs was around having hope to be healed. A male child said: “because they’ve given me medicine, I can recover, and I can return home” and a female child said: “when I come, and I need medication and I receive medication, I have hope”. This included the desire to return to school after receiving treatment. One female child said, “I will go to school next year and talk to them” while another said: “I wish to study, to become a teacher”.

A final aspect that emerged around resilience among female young adults was having staff that cared for them well. A female young adult said “staff in here are working well, they treat me well, and medication is fine. So, we thank them”.

## Discussion

This study is, to the best of our knowledge, the first to identify cancer-related concerns and needs of care and support among young adults and children who are receiving cancer treatment in Dar es Salaam, Tanzania. The concerns expressed include physical effects of cancer, emotional effects of cancer, financial impacts, poor early cancer care, and poor cancer treatment. The identified needs include the need for improved care in hospital by the staff, need for community support, financial needs, needs for improved cancer care and treatment in the hospital, and the need for increased education about cancer. Resilience was identified, particularly around hope or faith, having hope to be healed, and receiving good care from staff.

Cancer is known to take a toll on young adults and children physically [26, 27], emotionally [26, 27], and financially [26, 28], which was also evident for Tanzanian young adults and children. Pain and side effects of medication and treatment is an ongoing concern for many children and adolescents in other settings [29, 30], similar to the findings of this study. The current study found that children were affected emotionally, they felt separated from family members, and had negative feelings related to diagnosis. This finding has been shown for other cancer populations [27, 31]. They were also affected financially, despite that cancer treatment is supposed to be free in Tanzania after diagnosis, participants expressed that it was not. This is a challenge in a country such as Tanzania where many residents are unable to pay the costs associated with cancer treatment. Interestingly, the male young adults but not the other groups mentioned that they had to pay bribes to receive treatment. In a study on corruption on cancer-related healthcare in Africa, issues with accessing drugs and medical equipment as well as informal payment was found to be an issue in Tanzania [32]. This is a huge challenge for this vulnerable population and in order to address the needs of young adults and children receiving cancer treatment in Tanzania, it is essential that the issue of bribery is addressed by the responsible authorities.

Not surprisingly, many of the concerns expressed by participants were also reflected in the mentioned needs for care and support. Participants expressed concerns around poor early cancer care associated with wrong diagnosis and/or treatment leading to multiple visits to the healthcare before a correct diagnosis was made. Similar problems have been found elsewhere in Africa. A qualitative study using photovoice in South Africa identified challenges regarding poor services including need to travel far distances for treatment, poor care in hospital, and delay in cancer diagnosis [26]. However, due to a limited number of healthcare facilities providing cancer-related care and treatment in Tanzania, many cases are not diagnosed until late. Late stage presentation of cancer not only decreases the odds of survival but also puts a significant burden on the healthcare system [26]. Improved education about early signs of cancer and early diagnosis would increase chances of survival. Increased number of cancer centers as well as improved care in those centers could help address the concerns and needs of young adults and children receiving cancer treatment. The findings from this study indicate a need for a policy dialogue by the Ministry of Health, Community Development, Gender, Elderly and Children to enhance diagnosis and management of cancer in Tanzania.

Participants expressed concerns around the treatment they received while in the hospital. Not only was treatment and medication not always available, participants also expressed that machines were often broken or not working, and that they received poor care from the hospital staff. Accordingly, they expressed needs for better care from staff and improved support from government to fund hospitals with medication and equipment. They also expressed a desire for more local cancer centers; currently there are only five cancer centres in Tanzania, two located in Dar es Salaam and one each in Mwanza, Kilimanjaro, and Mbeya, all of which are at far distances from the rural areas with limited services. Tanzania currently has a limited number of healthcare providers and similar to other African countries, the burden of other diseases such as malaria, HIV/AIDS, and tuberculous place a significant strain on the healthcare system [33].

Participants expressed resilience in the face of their cancer care treatment. Faith provided significant support during treatment, as well as hope felt when receiving treatment and positive care from hospital staff. This finding support the previous research showing that faith can act as a protective factor during cancer treatment [34, 35]. For instance, Lagman et al. [34] found that Filipina immigrant breast cancer patients used faith to cope with the stress of diagnosis and treatment through prayer for themselves, prayer by others, and support from their belief community. Thus the findings indicate that faith, can complement community support, financial and emotional support to help coping with cancer, as proposed in a literature review on teenagers and young adults during cancer treatment [36].

## Limitations

We employed FGDs with both children and young adults which gave us an opportunity to gather collective and in-depth experiences of the participants. Through moderation processes, participants, particularly young adults, were able discuss and agree on their common as well as differing experiences, concerns and needs. The interactive debate, however, was less evident among younger children, who seldomly disagreed to peers’ ideas, and instead they focused more on sharing their views based on their personal experiences [37]. Nevertheless, active facilitation by the moderator through follow up questions to other participants to check if they agree or had the same experience on what was said by their fellow participants, helped to keep the group interactive.

Another limitation was that the transcripts of the audio-taped FGDs were translated from Kiswahili into English for the purpose of analysis which potentially could have resulted in loss of important data. However, to minimize this risk, the Kiswahili speaking co-authors GM and TK reviewed the transcripts to ensure the data were reported correctly and that the categories that emerged in the analysis were in accordance with the original Kiswahili audio-recordings. Also, it could be argued that different interview-questions should have been posed to young adults and children respectively. However, we chose to formulate questions that could be used by both age-groups and the participants’ answers to the questions did not indicate that there were any problem for either age-group to understand and answer the questions.

## Conclusion

This is the first study to explore cancer-related concerns and needs among young adults and children on cancer treatment in Tanzania. Participants expressed emotional, physical, and financial concerns as well as concerns around early cancer care and cancer treatment at the hospital. Needs of improved cancer care and treatment in the hospital, need for community support, financial needs, and the need for increased education about cancer were identified. Despite many concerns and needs, participants expressed resilience. As cancer rates continues to increase in Tanzania, the suggested improvements may be necessary to meet the concerns and needs of young adults and children currently receiving treatment. Further studies on specific approaches to address the concerns and needs are also warranted.
